# Perfluorocarbon Nanoparticles Loaded with Oxygen Alleviate Acute Kidney Injury via Ameliorating Renal Oxygenation Level

**DOI:** 10.34133/bmr.0181

**Published:** 2025-04-10

**Authors:** Dasheng Li, Yisong Ju, Qingsong Ye, Yuanyuan Chang, Chaoli An, Beibei Liu, Li Lu, Jinhui Wu, Xiaozhi Zhao

**Affiliations:** ^1^ Department of Andrology, Nanjing Drum Tower Hospital, The Affiliated Hospital of Nanjing University Medical School, Nanjing, Jiangsu 210008, China.; ^2^ Department of Urology, Nanjing Drum Tower Hospital, The Affiliated Hospital of Nanjing University of Chinese Medicine, Nanjing, Jiangsu 210008, China.; ^3^ Department of Andrology, Nanjing Drum Tower Hospital, The Affiliated Hospital of Nanjing University of Chinese Medicine, Nanjing, Jiangsu 210008, China.; ^4^ State Key Laboratory of Pharmaceutical Biotechnology, Medical School of Nanjing University, Nanjing 210093, China.

## Abstract

Renal microcirculatory disturbances and tissue hypoxia play a pivotal role in acute kidney injury (AKI) initiation and progression, and addressing renal hypoxia during the acute phase presents a promising therapeutic strategy for preventing AKI or protecting kidney function. In this study, we explored the renal protective potential of perfluorocarbon nanoparticles (PFPs), engineered for superior oxygen-carrying and delivery capacities, in an AKI mouse. Specifically, PFP-treated mice exhibited significant reductions in tubular dilation, necrosis, and brush border loss in renal tubules. Additionally, PFP pretreatment reduced tissue inflammation and fibrosis, as indicated by decreased nuclear factor-kappa B, α-smooth muscle actin, fibronectin, and collagen I expression. Serum creatinine and blood urea nitrogen levels improved, decreasing by 26.9% and 41.7%, respectively. Flow cytometry further showed controlled levels of f4/80^+^ macrophages and CD45^+^ inflammatory markers, with f4/80^+^ macrophages reduced by approximately 31.2% and CD45^+^ inflammatory factors reduced by 40.5%. Metabolomic analyses highlighted PFP’s modulation of key metabolic pathways linked to renal recovery, notably up-regulating slc22a19 by 48.3%, a gene encoding a short-chain fatty acid transporter, and down-regulating hyaluronic acid synthesis in renal tissue. These findings are the first to demonstrate that PFPs, as an oxygen carrier, can enhance renal resilience against IR (ischemia–reperfusion)-induced AKI, offering compelling evidence of PFP’s clinical potential in AKI management.

## Introduction

In the domain of cardiac and major vascular surgeries, as well as renal procedures, there is an augmented vulnerability to renal ischemia–reperfusion (IR) injury (IRI), which can precipitate acute kidney injury (AKI). AKI is characterized by a precipitous decline in renal function, typically manifesting within hours to days, and has emerged as a notable global health challenge [1]. Annually, there are over 5,000 cases of AKI per million people, with approximately 2 million deaths attributed to the condition annually [2,3]. Clinical diagnosis of AKI is predicated on the biochemical evidence of elevated serum creatinine (Cr) levels or diminished urinary output. The literature affirms the benefits of a comprehensive perioperative strategy for renal protection, including risk assessment, hemodynamic management, and postoperative surveillance, which collectively aim to reduce renal IRI and preserve kidney function [4]. Interventions like renal protective ventilation and targeted pharmacological agents that mitigate inflammation and oxidative stress have shown efficacy in lowering AKI incidence. Ischemic preconditioning also holds promise for enhancing renal cell tolerance to ischemia [5,6]. Despite progress, the lack of a specific AKI treatment remains a challenge, highlighting the need for further research to understand its pathophysiology and develop effective therapies.

Renal pathology can ensue from diverse etiologies, including ischemia, exposure to nephrotoxins, dehydration, or sepsis. Perturbations in the intrarenal microcirculation and tissue oxygenation are regarded as fundamental pathophysiological mechanisms in the genesis of AKI [7–10]. These mechanisms not only predominate during the acute phase of renal insult but also are instrumental in the progression to chronic kidney disease (CKD). Consequently, augmenting renal oxygen supply and optimizing intrarenal oxygen distribution may present a viable therapeutic strategy in the management of AKI. Current renal preservation strategies are primarily directed at reestablishing hemodynamic equilibrium. For instance, the employment of vasopressors such as metaraminol has been demonstrated to restore blood pressure and augment renal perfusion, particularly to the renal outer medulla [11]. However, it has shown limited advancement despite promising preclinical findings.

Perfluorocarbons (PFCs), celebrated for their exceptional oxygen affinities, biological inertness, and established safety profiles, have become a focal point of rigorous scientific inquiry [12]. PFCs have been harnessed to enhance oxygen and CO_2_ permeability in bioreactors and are under exploration for their therapeutic potential in conditions characterized by prolonged tissue ischemia. Notably, PFC-based oxygen therapeutics, exemplified by ABL-101, have demonstrated promise in clinical trials for the treatment of acute ischemic stroke [13]. These trials are capable of monitoring the metabolic status of tissues under oxidative stress to delineate salvageable tissue zones while concurrently reducing the ischemic impact through enhanced oxygen delivery [14,15]. However, the application of PFCs in the treatment of AKI mediated by IRI, with a focus on the amelioration of renal oxygenation, remains a relatively unexplored frontier.

This research introduces a pioneering application of PFC nanoparticles in the therapeutic arsenal against AKI. Capitalizing on the distinctive attributes of PFCs, such as their exceptional oxygen solubility and biocompatibility, this study offers an innovative modality for enhancing renal resilience against ischemic episodes in the perioperative setting. This novel synthesis has the potential to refine AKI treatment strategies, providing a more efficacious and targeted approach to renal protection and recovery.

In the field of AKI treatment, exploring innovative strategies to alleviate renal ischemia and oxidative stress is essential [16,17]. Perfluorocarbon nanoparticles (PFPs), with their high oxygen solubility, hydrophobicity, and low reactivity, offer a promising alternative for enhancing renal oxygenation and countering hypoxia-induced reactive oxygen species (ROS) production. This study aimed to evaluate the efficacy of PFP-based oxygen carriers in preserving renal function and reducing renal injury following ischemic events, while also assessing PFPs’ potential to improve AKI clinical outcomes by modulating oxidative stress and inflammation. This approach could provide a cost-effective, patient-centered strategy for AKI treatment.

## Materials and Methods

### Materials

The fetal bovine serum (FBS), Dulbecco’s Modified Eagle Medium/Nutrient Mixture F-12 (DMEM/F12) medium, and Roswell Park Memorial Institute (RPMI) 1640 medium were from Gibco (Grand Island, USA). Insulin–transferrin–selenium (ITS) was from Invitrogen (Carlsbad, CA, USA), and mouse epidermal growth factor (mEGF) was provided by R&D Systems (Emeryville, CA, USA). 2′,7′-Dichlorofluorescein diacetate (DCFH-DA), RIPA lysis buffer, and BCA Protein Assay Kit were from Beyotime (Shanghai, China). Phosphatase and protease inhibitors were provided from Boster (Wuhan, China). The Annexin V–APC/PI Apoptosis Detection Kit was from Yeasen (Shanghai, China). Hematoxylin and eosin (H&E) was from Servicebio (Wuhan, China). Diaminobenzidine (DAB) was from ZSGB-BIO (Beijing, China). The antibodies used for immunohistochemistry (IHC) were as follows: fibronectin (cat. no. ab2413, Abcam, Cambridge, MA, USA), α-smooth muscle actin (α-SMA, Proteintech, Wuhan, China), and collagen I (cat. no. ab34710, Abcam, Cambridge, MA, USA). Cr and blood urea nitrogen (BUN) kits were from Adsbio (Jiangsu, China). H&E was from Servicebio (Wuhan, China). Periodic acid–Schiff (PAS) was from Ruchuang (Shanghai, China). TdT-mediated dUTP Nick End Labeling (TUNEL) BrightGreen Apoptosis Detection kit was purchased from Roche (Shanghai, China). Fluorescein isothiocyanate (FITC) anti-mouse CD11b, PE anti-mouse Ly6G, and PerCP anti-mouse CD45 were provided by Thermo Fisher (Shanghai, China). Mouse interleukin-1β (IL-1β) ELISA (enzyme-linked immunosorbent assay) Kit (cat. no. FMS-ELM002, Nanjing, China) and Mouse IL-6 ELISA kit (cat. no. FMS-ELM006, Nanjing, China) were provided by the FCMACS (Nanjing, China). HK-2 and NRK-52E were provided by the Cell Bank of Shanghai Institutes for Biological Sciences (Shanghai, China). Perfluoro-n-pentane was provided by Baiduchem (Hubei, China). Egg yolk phospholipids were provided by Ukang Fly Company (Xian, China). Medium-chain triglyceride oil was provided by Lefu biotech company (Shenzhen, China)

### Synthesis of PFPs

PFPs were synthesized via unfolding/self-assembly technique. Briefly, in a 5-ml PFP emulsion, the composition includes 2% (w/v) egg yolk lecithin, 100 μl of medium-chain triglyceride, and 100 μl of perfluoropentane. These three components were combined and subjected to emulsification using a cell ultrasonic disruptor at 60% power with a cycle of 2 s on and 3 s off, for a total duration of 4 min. The PFPs exhibited a uniform spherical morphology, as observed through a scanning electron microscope.

### Dynamic light scattering measurements

Dynamic light scattering (DLS) also known as photon correlation spectroscopy or quasi-elastic light scattering, measures the very small relative frequency shift of scattered light. PFP nanoparticles (100 μl) were prepared and injected into 5 ml of deionized water, followed by thorough mixing. The DLS measurements were performed using the NanoBrook 90plus PALS, which is capable of providing accurate particle diameter distribution and hydrodynamic diameter measurements. The measurements were conducted at a controlled temperature of 25.00 °C to ensure stability and accuracy. The scattering angle used was 90.0°, which is optimal for detecting the size distribution of nanoparticles in the given medium. The polydispersity, count rate (kcps), and baseline index of the medium were carefully measured and recorded as 0.115, 429.5, and 9.4, respectively, to ensure accurate interpretation of the results.

### Extraction and culture of primary renal tubular epithelial cells

Kidneys from young mice less than 1 week old were harvested under sterile conditions and placed in prechilled saline. The kidney tissue was then homogenized, filtered through a 50-mesh and subsequently a 150-mesh sieve, and centrifuged at 1,000 *g* for 5 min to discard the supernatant. To the pellet, DMEM/F12 was added, and the mixture was incubated at 37 °C with intermittent shaking every 5 min for 25 min to facilitate cell dissociation. Following this, the cells were centrifuged at 1,000 *g* for 5 min to remove the supernatant. Then, complete DF12 medium, supplemented with 10% FBS, 1% penicillin and streptomycin, 1% ITS, and 10 ng/ml mEGF, was added to the cells. The primary tubular epithelial cells (TECs) were then evenly distributed and seeded into a cell culture dish. After 24 h, the medium was replaced. Cells reaching 80% to 90% confluence were utilized for subsequent experiments.

### Hypoxia-reoxygenation cell model

Primary mouse kidney cells (8 × 10^6^) were seeded in a 60-mm-diameter Petri dish and allowed to fully attach. PFPs were added to the culture medium at a final concentration of 10 nM, and the cells were subjected to a 12-h pretreatment. Following pretreatment, the medium was replaced with serum-free DMEM. The hypoxia chamber was prepared by introducing an oxygen-depleting agent and an oxygen indicator. The chamber’s seal and oxygen levels were verified prior to use. During the incubation, within approximately 10 min, the lid of the hypoxic chamber will be seen to be recessed. After about 20 min, the oxygen indicator will change from blue to pink, indicating that the oxygen level in the hypoxia chamber has dropped below 0.1%. Cells were subjected to hypoxic conditions for 2 h, after which they were transferred to an incubator with normal oxygen levels for 3 h of reoxygenation before being collected for downstream assays.

### Detection of ROS

ROS activity was measured using the ROS Assay Kit as directed by the manufacturer’s instructions. After washing cells with DMEM/F12 medium, cells were incubated with the ROS probe DCFH-DA for 20 min at 37 °C in a CO_2_ incubator. Post incubation, cells were washed again, and ROS production was assessed using a fluorescence microscope and pictures were taken.

### Flow cytometry for cell apoptosis assessment

To assess apoptosis in TECs, we used the Annexin V–FITC/PI Apoptosis Detection Kit in combination with flow cytometry. Cells were collected via centrifugation and resuspended in phosphate-buffered saline (PBS) following apoptosis induction. The Annexin V–APC and propidium iodide (PI) staining solution was added to the cell suspension, followed by a 15-min incubation at room temperature in the dark. After incubation, the cells were kept on ice prior to flow cytometry analysis. Annexin V–APC was detected using a 488-nm excitation wavelength and a 525-nm emission wavelength, while PI was detected with a 535-nm excitation wavelength and a 615-nm emission wavelength.

### Animals’ preparation

Mice were maintained under specific pathogen-free conditions at the Laboratory Animal Center of Nanjing University. All animal experimental protocols were approved by the Institutional Animal Care and Use Committee of Drum Tower Hospital, Medical School of Nanjing University, and were conducted in accordance with the institution’s guidelines. Male C57BL6 mice (SPF grade, 6 to 7 weeks old, 20 to 25 g) were sourced from Nanjing Qing Longshan Animal Farm.

### Animal models

An IR-induced AKI model was established in mice anesthetized with 5% pentobarbital. Throughout the procedure, mice were kept in a water bath with a core body temperature of 37 °C. Unilateral renal ischemia was induced by clamping the left renal pedicle. After 50 min of ischemia, the clamp was released to allow reperfusion, confirmed by the renal blood flow to its original color. Control group mice were preoxygenated through a mask for 30 min at a flow rate of 5 l/min. The identification group received an intravenous injection of 50 mg/kg PFPs without any gas, while the treatment group was administered with 50 mg/kg PFPs prefilled with oxygen, both 30 min prior to the procedure. The control group mice were given oxygen through a mask 30 min in advance, with a flow rate of 5 l/min, and sufficient oxygen was inhaled. After inhalation, an IR-induced AKI mouse model was created. The treatment group mice were infused with 50 mg/kg PFPs (with sufficient oxygen) 30 min in advance. We define the experiment group as IR, the blank group as Sham, and the treatment group as Treat.

### In vitro O_2_ absorption and depletion

After adding oxygen containing PFPs and oxygen-rich water to hypoxic and normoxic water, the O_2_ content mediated by PFPs was measured in vitro using an O_2_ microelectrode, and the kinetic curve of oxygen concentration was detected. In short, add 1 ml of water to the small bottle, cover it with mineral oil to form an airtight seal, and monitor the oxygen concentration for 10 min before and after laser irradiation. Among them, “normoxic” refers to 20% to 21% oxygen under atmospheric conditions, while hypoxic water achieves hypoxic conditions by deoxygenating water with vitamin C.

### BOLD-MRI image analysis

Blood oxygen level-dependent magnetic resonance imaging (BOLD-MRI) is an functional magnetic resonance imaging (fMRI) technique that uses deoxyhemoglobin as an endogenous marker to indirectly reflect the oxygen content in tissues. The R2* value, reflecting tissue deoxyhemoglobin content, was used to evaluate changes in renal oxygenation. After the decrease of tissue oxygen content, the content of deoxyhemoglobin increases, resulting in the decrease of effective transverse relaxation time (T2*) value, and the increase of apparent transverse relaxation rate (R2*; R2*=1/T2*). Therefore, the R2* value is positively correlated with the content of deoxyhemoglobin in the tissue, which can indirectly reflect the oxygen content of kidney tissue [18,19].

In reference to the BOLD images, select kidney skin and medullary demarcation, and compare good images. In at least 4 levels of anterior and posterior, centered on the renal hilum, areas where the skin and medulla are clearly distinguished at the upper, middle, and lower poles of the bilateral kidneys, ROI was placed in the renal cortex and medulla region. Try to avoid the renal sinuses and vascular structures. The size is approximately 18 to 22 mm^2^. Ensure the same shape and size of ROI in different parts of the same patient; R2* values for at least 24 ROI were measured in the renal skin, medulla region. The average R2* values of the skin and medulla were calculated and recorded.

### IHC and histology

Kidney sections were processed through deparaffinization rehydration and antigen retrieval using sodium citrate buffer. After blocking with 5% goat serum albumin, sections were incubated with anti-fibronectin and anti-collagen I antibodies overnight at 4 °C. Following incubation with a secondary antibody, the sections were stained with DAB chromogen and counterstained with hematoxylin. In addition, H&E staining was performed to evaluate inflammatory cell infiltration. Slides were examined under a fluorescence microscope, and immunoreactivity was quantified using Image-Pro Plus v.6.0 software.

### Enzyme-linked immunosorbent assay

Mouse IL-4 and IL-6 ELISA Kits were utilized according to the manufacturer’s instructions. Samples and standards were pipetted and added into a 96-well plate, followed by the addition of a horseradish peroxidase-labeled detection antibody. After incubation at 37 °C and washing, the microplate working solution and chromogenic agent were added sequentially. The absorbance at OD_450_ (optical density at 450 nm) was measured after the addition of the stop solution.

### Cr and BUN testing

Serum Cr and BUN levels were assessed in mouse serum samples. Standards and samples were added to a detection plate, and after the addition of the detection antibody and incubation, the plate was washed. The substrate reaction was allowed to proceed before the stop solution was added, and absorbance at OD_450_ was detected.

### TUNEL detection

TUNEL was performed to detect DNA fragmentation. Paraffin sections were deparaffinized, hydrated, rinsed with PBS, permeabilized with proteinase K, and labeled with fluorescein-dUTP using terminal deoxynucleotidyl transferase. After being stained with nuclei and mounted, slides were visualized under a fluorescence microscope within a wavelength range of 450 to 500 nm.

### Measuring oxygen reserve capacity

To measure oxygen reserve capacity, we used an O₂ microelectrode for both in vivo and in vitro experiments. The microelectrode was inserted into the renal cortex of mice for in vivo monitoring of oxygen content changes. The specific details of the oxygen electrode are as follows: manufacturer: Unisense, Denmark; model: O₂ Microsensor OX-10 standard; sensitivity: <2%; response time: 1 to 3 s; measurement temperature: 20 °C; salinity: 0. The O₂ microelectrodes were purchased from Unisense, a world-leading manufacturer of high-performance microsensors, with a linear response range from 0% to 100% O₂ and optimized sensitivity for detecting dissolved oxygen. The electrodes were calibrated using a 2-point calibration method with standard oxygen solutions to ensure accurate and reliable measurements across the entire range of oxygen concentrations.

### Statistical analysis

GraphPad Prism 8 was used for statistical analysis. Data are presented as mean ± standard error of the mean (SEM) or standard deviation (SD). Student’s *t* test was applied for pairwise comparisons, while one-way analysis of variance (ANOVA) with the Newman-Keuls Multiple Comparison Test was used for multiple group comparisons. A *P* value of <0.05 was considered statistically significant.

## Results

### Characterization of nanoparticles and monitoring of O_2_ reserve and release levels of PFPs

**Fig. 1.**
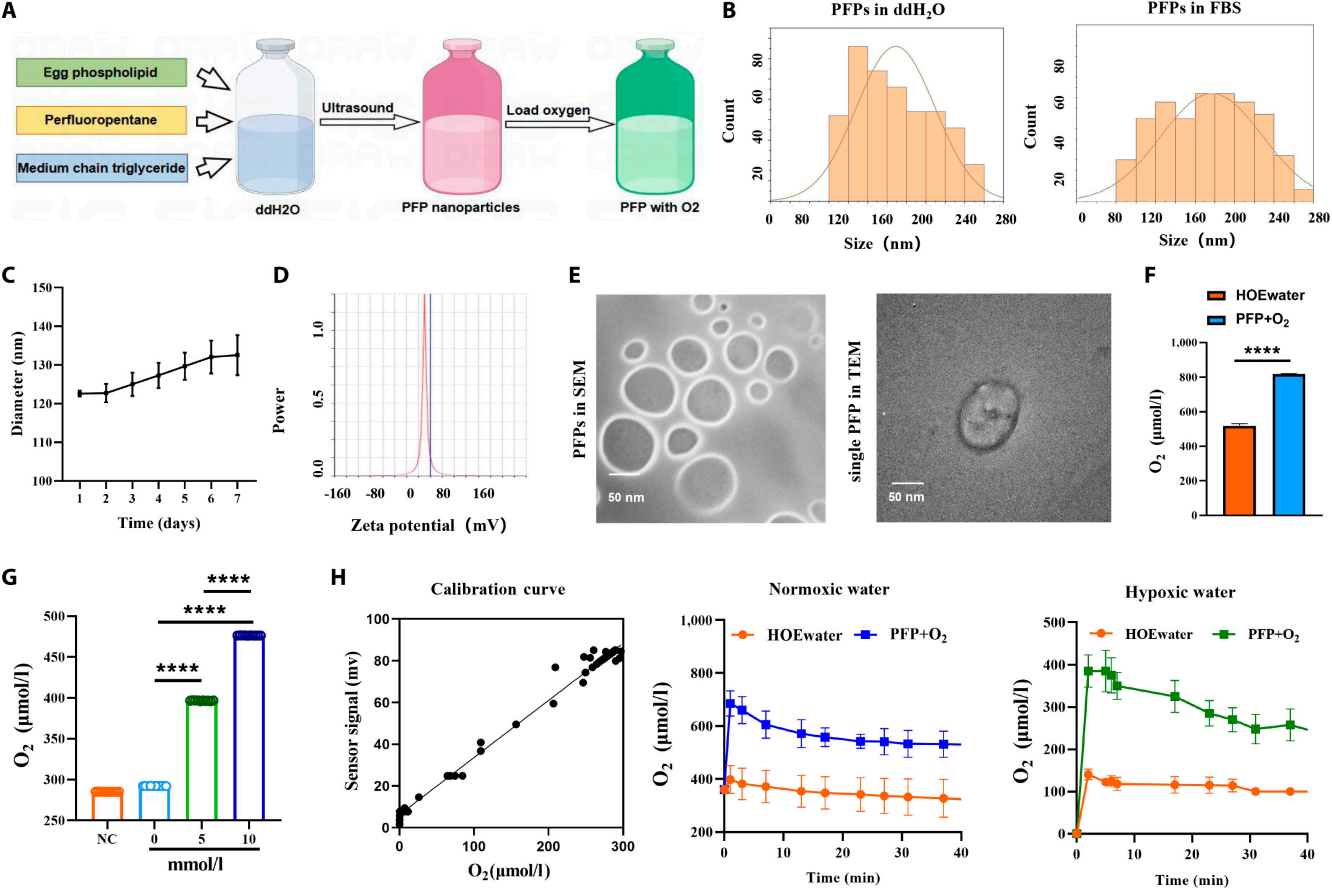
PFP characterization and oxygen reserve and release analysis. (A) Schematic representation of PFP synthesis. (B) Particle diameter distribution of synthesized PFPs as determined by DLS in ddH_2_O (left) or FBS (right). (C) The particle diameter of PFPs in PBS over 7 days is basically stable. (D) Zeta potential measurements indicate the surface charge of PFPs. (E) TEM and SEM micrographs illustrating the morphology of PFPs. (F) Comparative analysis of dissolved oxygen content between oxygen-loaded PFPs and HOE water (*n* = 5). (G) Oxygen release profiles of PFPs at varying concentrations in deoxygenated water. (H) Calibration plot resulting from the plateau currents measured at 0.0 V as a function of the O_2_ concentration measured in solution. Kinetic curves of oxygen concentration changes following the addition of oxygen-loaded PFPs and oxygen-rich water to normoxic water and hypoxic water (*n* = 10). Data are presented as mean ± standard deviation, with statistical significance determined by a 2-tailed *t* test (****P < 0.0001).

PFPs were fabricated via a novel unfolding/self-assembly technique pioneered by our research consortium. The synthesis process integrated the phacoemulsification of 2% (w/v) egg yolk lecithin, 100 μl of medium-chain triglyceride, and 100 μl of perfluoropentane, as depicted in Fig. 1A. PFP is a highly hydrophobic liquid with a density greater than water, while PFCs emulsion consists of small oil-in-water droplets uniformly dispersed and encapsulated by an emulsifier, similar to an intravenous fat emulsion. DLS analysis confirmed a uniform particle diameter of approximately 120 nm (Fig. 1B). To investigate the dispersion stability of PFPs in serum-containing solutions, we employed a PBS solution supplemented with 10% FBS to simulate the in vivo serum environment. This in vitro model allowed us to evaluate the colloidal stability of PFPs under conditions that closely mimic physiological settings. DLS measurements revealed that PFPs maintained high dispersion stability in the FBS-containing PBS solution, with minimal aggregation observed over the experimental duration. These results indicate that PFPs can effectively retain their colloidal stability even in the presence of serum proteins, which is essential for their potential biomedical applications (Fig. 1B). In our measurements, the particle size variation of the prepared PFP nanoparticles over a 7-day period was approximately 10 nm, suggesting that while the particles do not exhibit significant aggregation, there is a slight change in size over time (Fig. 1C). In aqueous solution, the nanoparticles exhibit Brownian motion, and each particle’s encapsulated PFP droplets have a density similar to water. Furthermore, the inherent negative charge of PFPs, as revealed by zeta potential analysis, points to their favorable absorption by human tissues (Fig. 1D).

The negative charge of PFPs is crucial for their effective interaction with inflamed tissues. In the context of AKI, the inflammatory response leads to an accumulation of positively charged proteins within the renal tissues [20]. This environment results in the generation of ROS and other harmful reactions that exacerbate tissue damage [21]. The negatively charged surface of PFPs allows them to be more readily absorbed by the inflamed tissues, thereby reducing tissue damage and improving therapeutic outcomes [22]. Moreover, the cell membrane typically has a negative potential inside and a positive potential outside. During inflammation, the permeability of cell membranes increases, leading to the loss of negative ions [23]. Negatively charged nanoparticles, such as PFPs, can help maintain cell membrane integrity and ensure an adequate potential difference across the membrane. This facilitates the effective removal of inflammatory factors and other damage mediators, thereby enhancing the therapeutic efficacy in AKI treatment. Transmission electron microscopy (TEM) observations delineated a uniform spherical morphology for individual PFPs (Fig. 1E).

Given the superior oxygen absorption affinity of PFCs over water, the PFPs were hypothesized to bolster renal auto-oxygenation, critical for tissue recovery post-IRI. The PFPs were capable of absorbing and releasing higher quantities of dissolved oxygen, thus supporting tissue recuperation. To assess the oxygen reserve capacity of the PFP formulation, deoxygenated water was treated with nitrogen and then supplemented with varying concentrations of PFPs. The oxygen-enriched PFPs resulted in a marked increase in dissolved oxygen levels, surpassing that of heavy oxygen-enriched water (HOE water), a proxy for oxygen therapy, thereby highlighting the substantial oxygen reserve capacity of the PFP solution (Fig. 1F). A direct linear relationship was observed between PFP concentration and oxygen release, with higher concentrations correlating to increased oxygen content and release efficiency (Fig. 1G). Subsequently, an oxygen-saturated PFC solution and oxygen-rich water were introduced to normoxic and hypoxic water samples, respectively. Oxygen electrode monitoring confirmed that PFPs rapidly elevated oxygen levels and release rates under both conditions, outperforming conventional oxygen inhalation methods (Fig. 1H).

### PFPs’ protective role in renal oxygenation following IRI in mice

**Fig. 2.**
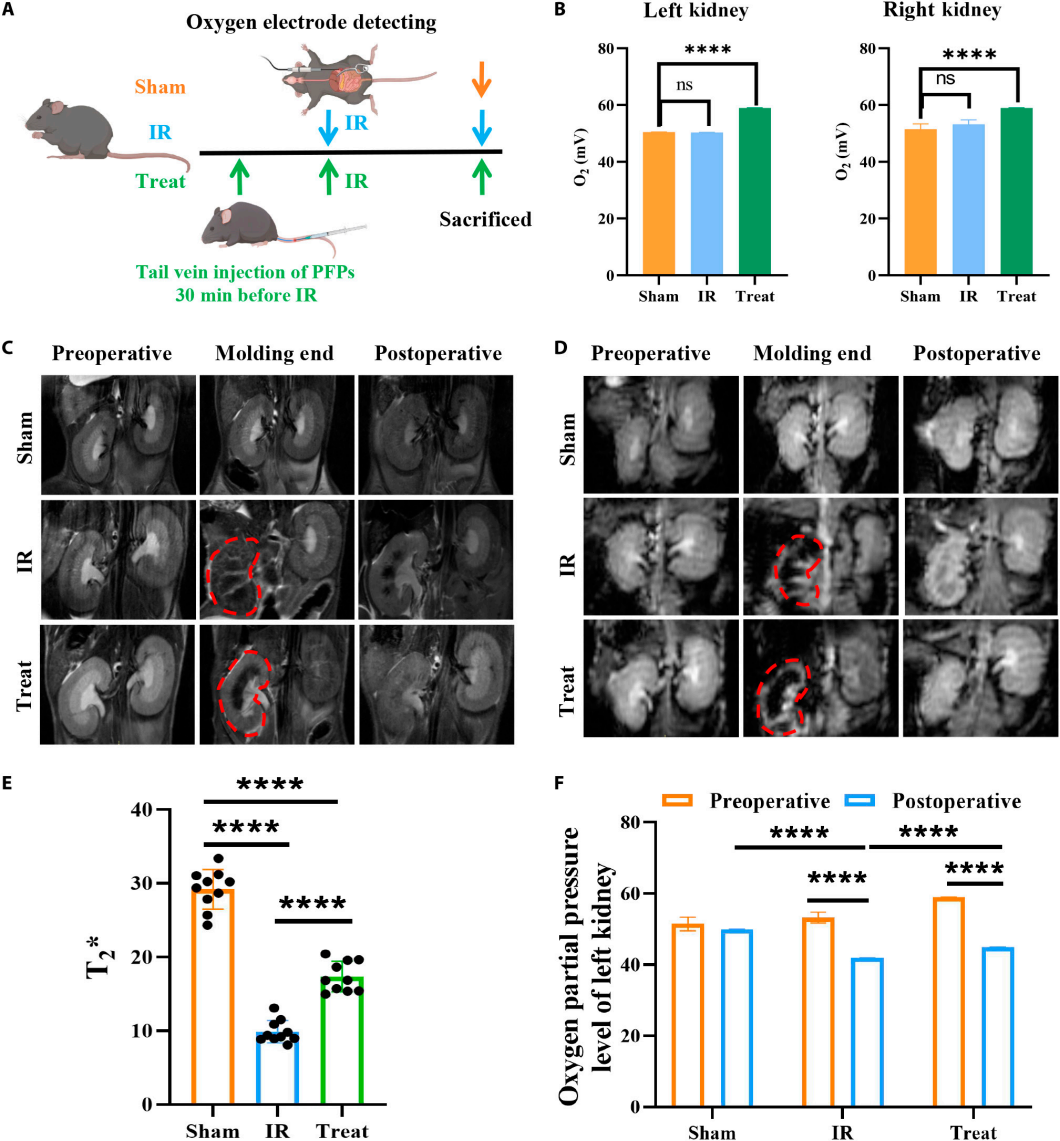
PFPs’ protective role in renal oxygenation following IRI in mice. (A) Schematic representation of the experimental setup, treatment protocols, and the methodology for monitoring in vivo oxygen concentration. (B) The renal tissue oxygen tension levels of the left and right kidneys measured using an oxygen electrode in different groups of mice prior to surgery to evaluate improvements in oxygen partial pressure (*n* = 10). (C) BOLD MRI T2*-weighted images depicting the progression of IR-mediated AKI in mice at various stages within different groups. (D) BOLD MRI analysis under the BOLD sequence illustrating the dynamic changes in different groups of mice subjected to IR-induced AKI. (E) Corresponding to 24 h after AKI induction, the ROI level of MRI images was quantitatively analyzed before kidney extraction for further analysis, reflecting the recovery of AKI in the experimental group (*n* = 10) mice. (F) Comparative analysis of oxygen partial pressure before and after left kidney surgery in each group of mice, as well as the difference in oxygen partial pressure between the IR group and the treatment group (*n* = 10). Data are presented as mean ± standard deviation. Statistical significance was determined using a 2-tailed *t* test (****P < 0.0001).

To elucidate the impact of PFPs on renal oxygenation in an in vivo AKI mouse model, we employed an oxygen electrode to assess the oxygen levels in the renal cortex of mice. The experimental design was structured to include an experimental group, a control group, and a treatment group. Oxygen levels were measured by inserting an oxygen electrode into the renal cortex, as depicted in Fig. 2A. In the initial phase of the study, we observed the oxygen partial pressure in the kidneys of the experimental group mice. Before the intervention, the oxygen partial pressure was measured in both the left and right kidneys using an oxygen electrode. The findings indicated that the oxygenation levels were comparable, suggesting that both kidneys were functioning normally and exhibited similar oxygenation capacities (Fig. 2B).

In the IR-induced AKI model, a significant decline in renal cortical oxygen levels was observed once the left renal pedicle was clamped by an arterial clamp, leading to ischemia. The cessation of oxygen partial pressure to zero confirmed complete ischemia of the left kidney. Upon release of the clamp, the recovery of oxygen partial pressure in the left kidney indicated the initiation of reperfusion. Concurrently, the right kidney exhibited no significant variation in oxygen partial pressure, maintaining a stable renal oxygenation level relative to the left kidney (Fig. 2C to E). Specifically, the T2* sequence reflects the signal intensity of intravascular blood flow, where higher signal intensity (brighter regions) indicates stronger blood flow, while lower intensity (darker regions) indicates weaker blood flow (Fig. 2C). In contrast, BOLD sequence is an advanced MRI technique that directly visualizes the oxygenation level of blood flow. In the BOLD sequence (Fig. 2D), higher signal intensity (brighter regions) corresponds to higher oxygenation levels, whereas lower intensity (darker regions) indicates lower oxygenation levels. By combining the T2* sequence (Fig. 2C) and the BOLD sequence (Fig. 2D), we were able to comprehensively illustrate dynamic changes in tissue oxygenation across different time points and experimental groups, allowing us to simultaneously assess both blood flow dynamics and tissue oxygenation status.

At the 24-h follow-up, we reassessed the oxygen content and found that revealing the oxygen partial pressure in the left kidney (the damaged side) had partially recovered but remained significantly lower than the presurgical baseline level (Fig. 2F). Through comparative analysis, we observed that in the IR-induced AKI model, the experimental group exhibited a significantly smaller decline in oxygenation levels after 24 h compared to the control group. Moreover, the treatment group showed a significantly higher oxygen partial pressure at 24 h post-surgery than the IR group, further confirming the effectiveness of preoxygenated PFPs in improving renal tissue oxygenation.

### PFPs inhibit hypoxia-induced injury in renal TECs

**Fig. 3.**
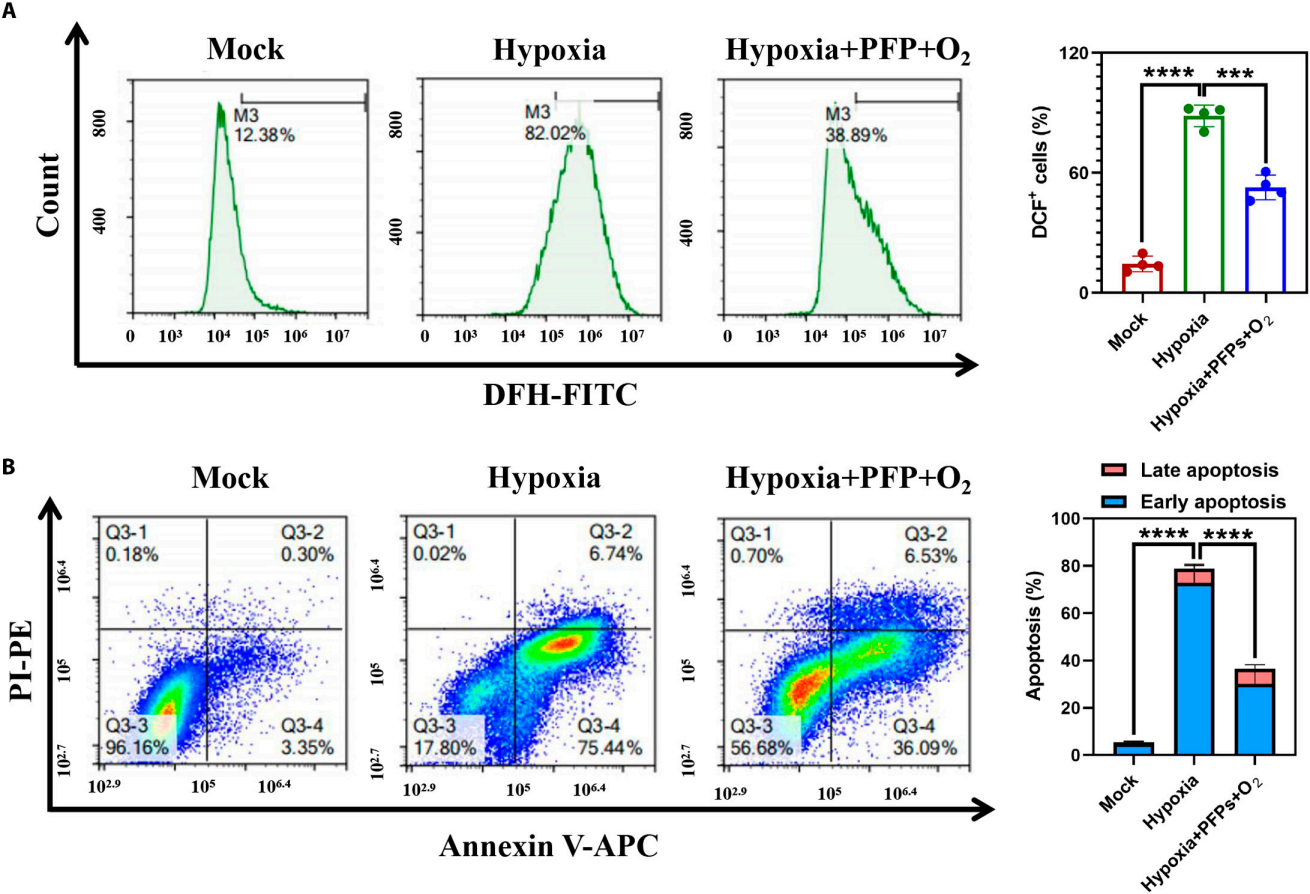
Exploring the therapeutic effect of PFPs. (A) Detection of ROS in renal TECs by preadding PFPs using flow cytometry (*n* = 4). (B) Flow cytometry was used to detect the in vitro anti-apoptotic activity of PFPs (*n* = 4). The data are the mean standard deviation, and the *P* value is a double-sided *t* test (***P < 0.001, ****P < 0.0001).

To elucidate the therapeutic efficacy of PFPs on hypoxia-induced renal TEC injury, cells subjected to hypoxic conditions were treated with PFPs, followed by flow cytometric analysis to quantify the levels of ROS and apoptosis. Our results revealed a pronounced decrease in ROS levels in the PFP-pretreated cells compared to the hypoxia-only group, indicating the ROS-scavenging capability of PFPs (Fig. 3A and Fig. S1A). The hypoxia-reoxygenation model was utilized to simulate IRI, with a final concentration of 10 μM PFPs employed for in vitro pretreatment. This treatment significantly ameliorated the apoptosis induced by hypoxia, as demonstrated by a marked reduction in the apoptotic index relative to the untreated control group (Fig. 3B). The underlying protective mechanism of PFPs is hypothesized to involve their antioxidant properties, which are suggested by the diminished ROS levels. Additionally, the observed reduction in apoptosis implies that PFPs may enhance cellular resilience to hypoxic stress, thereby maintaining cellular homeostasis.

### Therapeutic effect of PFPs in AKI mouse models

**Fig. 4.**
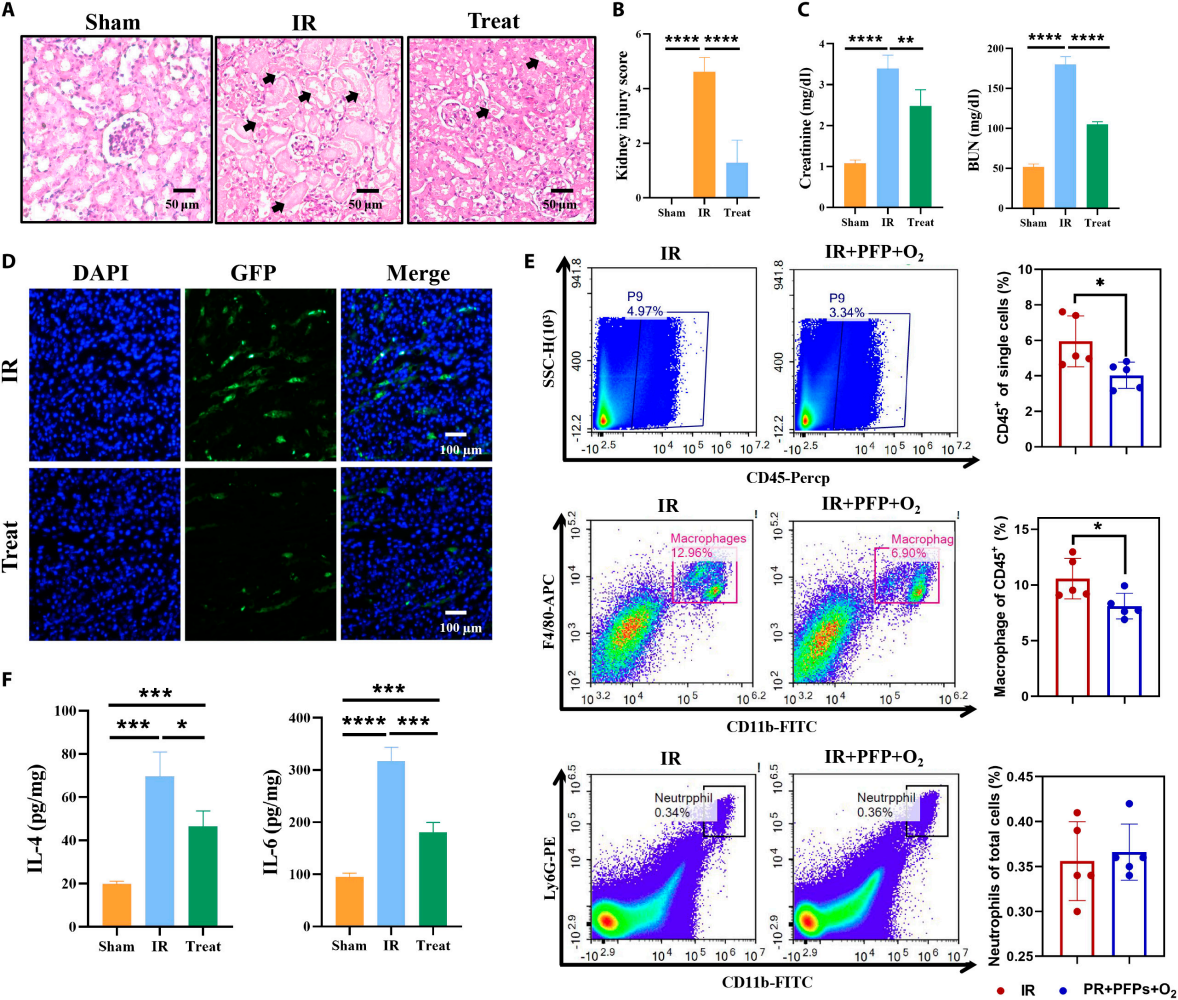
In vivo therapeutic effects of PFPs on AKI in mouse models. (A) Representative H&E-stained sections from different experimental groups, depicting renal morphology; the arrow indicates renal tubular injury, including obvious tubular necrosis, loss of brush like boundaries, and dilation (scale bar = 50 μm). (B) Quantitative comparison of renal injury scores among the groups based on H&E staining (*n* = 10 mice per group). (C) Comparative analysis of serological indicators between the experimental group and the PFP-treated group (*n* = 4). (D) TUNEL fluorescence staining illustrating the comparative levels of apoptosis between the experimental and PFP-treated groups. (E) Flow cytometry analysis of immune cell populations in peripheral blood and kidneys of mice, highlighting the modulatory effects of PFP treatment on immune cell subsets (*n* = 5). (F) Quantitative assessment of pro-inflammatory cytokines IL-4 and IL-6 levels in renal tissue lysates, comparing the experimental and PFP-treated groups. Data are presented as mean ± standard deviation (*n* = 5). Statistical significance was determined using a 2-tailed *t* test (*P < 0.05, **P < 0.01, ***P < 0.001, ****P < 0.0001).

To evaluate the therapeutic impact of PFPs on AKI in vivo, renal tissues were harvested 24 h post-AKI induction. H&E staining was utilized to scrutinize the morphological changes in the renal cortex. In comparison to the control group, intravenous administration of PFPs in normal mice did not induce significant pathological alterations to the glomeruli and renal tubules. In stark contrast, untreated AKI was characterized by severe tubular damage, including pronounced tubular necrosis, brush border loss, and dilation (black arrow). These histological manifestations were significantly mitigated following PFP treatment, as evidenced by a substantial reduction in the renal injury score (Fig. 4A and B). Further detection of serum biomarkers BUN and Cr for renal function revealed that PFP treatment significantly improved renal function damage in AKI mice (Fig. 4C). Additionally, the TUNEL assay demonstrated a marked decrease in the level of apoptosis within the renal tissue of the treatment group, suggesting that PFPs may exert a protective effect on renal cells by inhibiting apoptosis following IRI (Fig. 4D).

In the subsequent analysis, utilizing flow cytometry, we quantified the proportions of neutrophils, CD45^+^ cell subsets, and F4/80^+^ cell subsets in the kidney. A comparative analysis revealed significant alterations in the renal CD45^+^ and F4/80^+^ cell populations in untreated kidneys affected by AKI compared to the control group. Upon treatment with PFPs in the AKI mice, a pronounced reduction in renal CD45^+^ and F4/80^+^ cell populations was observed (Fig. 4E and Fig. S1B). Furthermore, both IL-4 and IL-6, recognized as key mediators of inflammation, exhibited a significant reduction in their levels post-PFP treatment, indicating a dampening of the inflammatory response in kidneys subjected to IRI (Fig. 4F). These findings suggest that PFP treatment modulates the inflammatory response in the renal tissues of AKI mice, potentially through the attenuation of specific leukocyte subsets.

### PFPs mitigate the progression of AKI to renal fibrosis

**Fig. 5.**
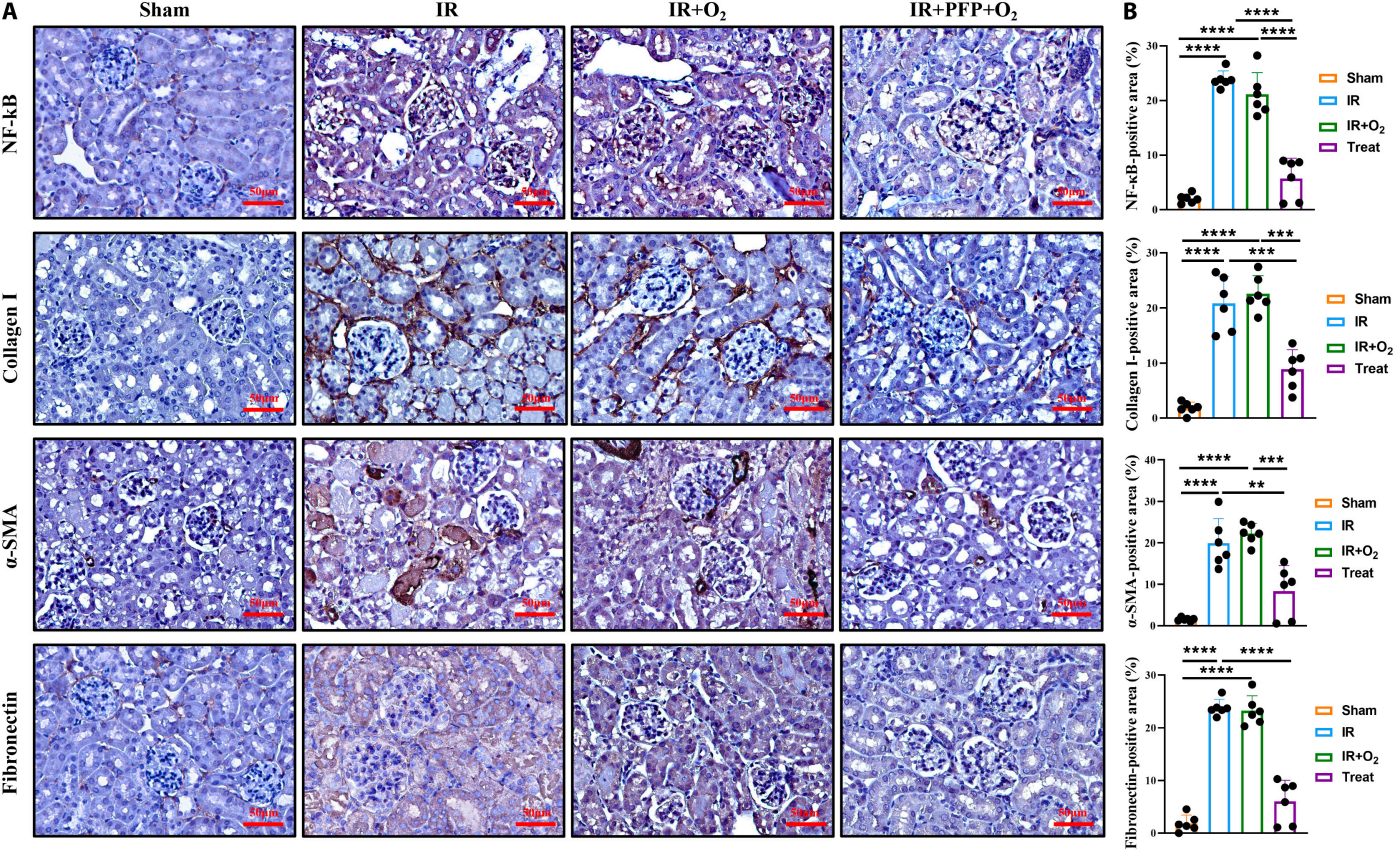
PFPs mitigate the progression of AKI to renal fibrosis. (A) Immunohistochemical staining of NF-κB, collagen I, and α-SMA and fibronectin in renal tissue slices between 3 groups; scale bar = 50 μm. (B) Positive tubular quantification of NF-κB, collagen I, and α-SMA and fibronectin staining. Statistical significance was determined using a 2-tailed *t* test (*n* = 6) (**P < 0.01, ***P < 0.001, ****P < 0.0001).

To elucidate the impact of PFPs on the inflammatory response in AKI and its potential to prevent the transition to renal fibrosis, immunohistochemical analysis was conducted to evaluate the expression of the inflammatory marker nuclear factor-kappa B (NF-κB) and fibrosis markers, including fibronectin, collagen I, and α-SMA. The extent of NF-κB expression was quantified by measuring the positive staining areas, revealing that PFPs significantly attenuated the up-regulation of NF-κB in renal tissues of AKI mice (Fig. 5A and B). Notably, the PFP-treated group exhibited a significant reduction in α-SMA, collagen I, and fibronectin compared to the other groups (Fig. 5A and B). A systematic comparison of the immunohistochemical staining across the groups was performed by quantifying the positive areas in representative images from each group. The analysis demonstrated a pronounced difference in the extent of fibrosis between the PFP-treated group and the experimental group, suggesting that PFPs may exert a protective effect against renal fibrosis progression in AKI (Fig. 5B).

### PFPs modulate short-chain fatty acids and hyaluronic acid content in mouse kidneys

**Fig. 6.**
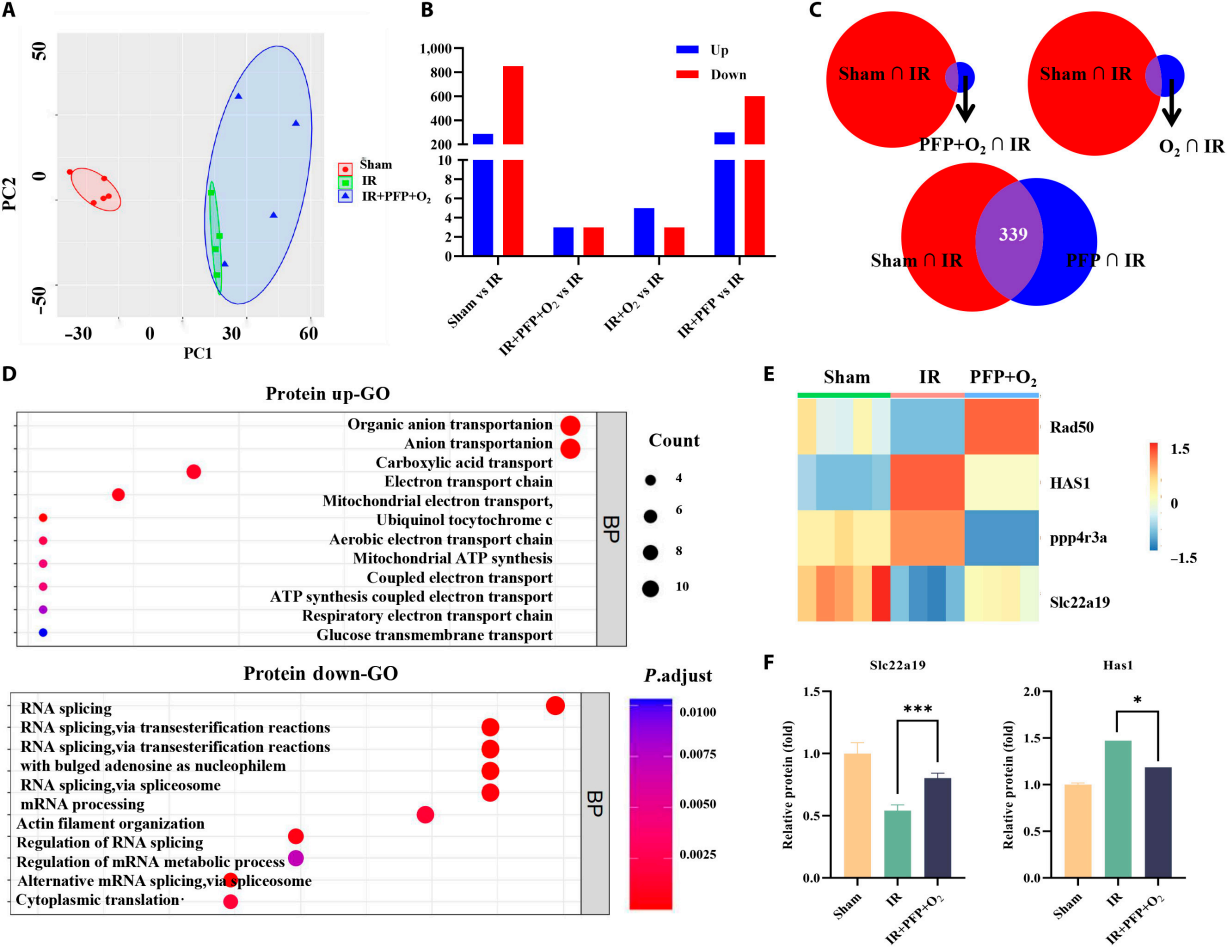
Transcriptomic analysis reveals differential gene expression patterns in mice subjected to various treatments. (A) Comparative transcriptomic profiles of renal tissues among different treatment groups (*n* = 5 mice per group). (B) Adjustment of the number of differentially expressed genes, highlighting the up- and down-regulation in response to treatment. (C) Venn diagram illustrating the overlap and uniqueness of differentially expressed genes across the normal, blank, control, identification, and experimental groups. (D) GO pathway analysis of the differentially expressed genes in mouse kidneys, providing insights into the biological processes affected by the treatments. (E) Heatmap depicting the expression profiles of renal genes associated with PFP therapy, visualizing the clustering of gene expression patterns. (F) Relative RNA expression levels of Slc22a19 and HAS1 genes in the kidneys. Statistical significance was determined using a 2-tailed *t* test (*n* = 4) (*P < 0.05, ***P < 0.001).

To elucidate the molecular mechanisms underlying the protective effect of PFPs in AKI, RNA sequencing analysis was performed on renal tissues from 5 distinct groups: normal, blank, control, identification, and experimental. Unsupervised principal component analysis revealed a clear separation of samples among the groups (Fig. 6A). Comparative gene expression analysis revealed that, relative to the blank group, the normal group exhibited 287 up-regulated and 851 down-regulated genes. Notably, although the identification group, which received PFPs injection alone, displayed a higher number of differentially expressed genes, the therapeutic impact was not statistically significant (Fig. 6B). Venn diagram analysis identified 339, 7, and 5 differentially expressed genes among the 3 groups when comparing the normal and blank groups to the control, identification, and experimental groups, respectively (Fig. 6C). Gene ontology (GO) enrichment analysis indicated that genes associated with anion transport, the electron transport chain, and ATP synthesis, including mitochondrial and aerobic transport proteins, were up-regulated. Conversely, genes involved in RNA splicing and mRNA processing were down-regulated (Fig. 6D).

Subsequent analysis focused on genes with differential expression patterns. A heatmap visualization demonstrated the regulatory roles of these genes in IR-mediated AKI and the therapeutic effects of PFPs (Fig. 6E). Among these, the solute carrier family 22-member 19 (slc22a19) gene and hyaluronan synthase 1 (HAS1), previously implicated in the acute phase and early recovery of AKI, were confirmed through Q-PCR analysis (Fig. 6F).

Short-chain fatty acids (SCFAs), as metabolites derived from the fermentation of indigestible polysaccharides by gut microbiota, are crucial for maintaining energy homeostasis [24]. Metabolic pathway analysis revealed that PFP treatment significantly up-regulated the SCFA-related transporter Slc22a19 in mouse kidneys, a finding corroborated by independent studies [25]. Additionally, the expression of HAS1, a marker of early injury in AKI, was found to be inhibited [26]. These observations suggest that PFPs may enhance SCFA levels and reduce HAS1 expression, which attenuate renal inflammation and injury.

## Discussion

AKI is a type of acute renal failure where there is currently no effective treatment except supportive care. Acute tubular necrosis, caused by renal IRI, and characterized by high morbidity and mortality, is the most common pathogenesis of AKI [27,28]. Renal IR causes microcirculation disturbances, while ischemia-induced renal hemodynamic changes reduce the oxygenation level of the kidneys [29]. It has been shown that renal hypoxia may facilitate the progression of kidney injury and hence increased proteinuria and renal fibrosis. Due to the uneven distribution of renal blood flow, the kidneys are particularly vulnerable to ischemic injury despite receiving nearly 20% of cardiac output, which triggers a low oxygen availability of the renal medulla [30]. As for ischemic renal injury, symptoms are normally seen, such as ischemia, ATP depletion, loss of cell adhesion, necrosis, and tubular obstruction. Notably, the restoration of renal blood flow during recovery is critical for long-term kidney function. The reperfusion, however, may trigger oxidative stress and inflammation and hence accelerate renal injury [31]. As for the renal IR-mediated AKI, current studies mainly focus on the clearance of ROS during reperfusion, while the direction toward the improvement of renal oxygenation level is rarely researched.

At present, as a Food and Drug Administration-approved product, PFPs have been widely adopted in fields such as biochemistry, where they increase the permeability of oxygen and CO_2_ in bioreactors. Applications have also covered the clinically artificial hemoglobin preparation and drugs for acute ischemic stroke [32–35]. However, it has not been formerly used for the treatment of AKI mediated by IRI. In this study, we employed the PFPs, characterized by high oxygen affinity, complete bio inertness, and safety, as the renal oxygen supply to improve intrarenal oxygenation levels.

PFPs have garnered remarkable attention due to their ability to dissolve substantial amounts of oxygen and carbon dioxide, along with their high hydrophobicity and low reactivity, allowing for efficient gas exchange at the tissue level [15,36]. In AKI, renal hypoperfusion results in tissue hypoxia, which triggers the release of excessive ROS, initiating an intense inflammatory response that ultimately degrades renal structure and function [28]. Alleviating hypoxia thus represents a promising strategy for AKI treatment. While hyperbaric oxygen therapy can balance oxidative and antioxidant systems by improving renal microcirculatory perfusion, its high cost and limited patient compliance hinder its widespread application [29]. Given the inert nature, small particle size, and high oxygen-carrying capacity of PFPs, we selected them as oxygen carriers to support renal function by ensuring sustained local oxygenation, thereby mitigating renal injury.

According to literature reports, PFC nanoemulsions have been used as artificial oxygen carriers and as renal protective solutions in kidney transplantation [37]. Additionally, PFPs have been employed as lung lavage fluids in the treatment of acute respiratory distress syndrome. With their low surface tension and anti-inflammatory properties, PFPs can effectively prevent alveolar collapse. They are currently being used in combination with other drugs in animal experimental studies [38].

To our knowledge, this is the first study to explore the therapeutic effect of improving the oxygenation level of the kidneys by infusion of PFPs filled with sufficient oxygen by preoperative pretreatment. The results showed that PFP products can antagonize IR-mediated AKI. Therefore, such a product is desired in a variety of disease states, including IRI, which wreaks havoc in tissue preservation and many other conditions where tissues undergo ischemia for prolonged periods.

In this study, we first explored the characterization of PFPs, including the validation of oxygen reserve capacity and oxygen release level in vivo and in vitro. By using hypoxia-induced TEC damage, it is found that PFPs can improve hypoxia, thereby reducing inflammatory factors and macrophage release to protect cells. In the IR-induced AKI mouse model, it is verified that PFPs help improve cast-forming, tubular necrosis, brush margin loss, and tubular dilation; alleviate renal damage; and have good renal protective effects. Additionally, PFPs may alleviate AKI by increasing the content of the short-chain fatty acid transporter slc22a19 and reducing the content of hyaluronic acid in mouse kidney tissue, as is verified in the metabolomic analysis. Therefore, the study provides a new perspective on the physiological treatment of AKI. We divided the animal model research groups into 5 groups in the preexperiment, namely, the blank group, the experimental group, control group 1, control group 2, and the treatment group. The blank group, the experimental group, and the treatment group were the same as the text content. Control group 1 was treated with oxygen mask inhalation at a flow rate of 5 l/min for 30 min before surgery, while control group 2 was treated with infusion of pure PFPs (without any gas) for 30 min before surgery. In the preexperiment results, the therapeutic effects of both control groups were minimal; thus, their effectiveness was not further verified in subsequent experiments. PFPs have a concentration-dependent oxygen storage and release capacity, and hence, their therapeutic effect should be increased with the volume of infusion. However, as the volume of infusion exceeds a certain threshold, experimental mice may die due to excessive volume load or other reasons. The concentration used in this study is the maximum value of the infusion volume that has the lowest mortality rate explored in the preexperiment. Its theoretical maximum therapeutic dose and optimal therapeutic effect may need further experimental verification. In addition, oxygen supply itself can increase the level of oxidative damage in the body so that possible impact can be observed on IR-mediated AKI in the detection of oxygen electrodes in vivo. After the modeling process, there is still a notable therapeutic effect in terms of oxygen levels and other related verifications. If the general treatment method of other AKI-related papers (drug infusion after modeling) is adopted, also considering the impact of oxygen, for common sense, it is not verified whether it can also play a therapeutic role. Further experimental verification may be needed for the above issues.

Notably, by improving the oxygenation level of the kidneys before surgery, this study mainly explores the capacity of PFP products to improve the treatment of AKI. However, compared with most AKI studies in recent years, whether PFPs can play a role in kidney targeting, and have a notable improvement effect on the kidney compared to other organs, has not been fully considered here. To reduce adverse effects and improve therapeutic efficacy, we need more clinical and preclinical data to help find the corresponding problems and further delve into the targeting of PFP products in the next generation. Additionally, renal arterial clipping is a common strategy in renal surgeries, such as kidney transplantation, and renal tumor resection, and clinical problems like AKI are hence induced in postoperative patients [39–42]. To tackle this, further clinical research and verification in clinical samples would be the promising direction. Specifically, improving the level of intraoperative oxygenation, the reduced probability of postoperative AKI, and improved recovery effect of postoperative AKI would be the goals for clinical applications. Future research into clinical translation would help to improve postoperative oxidative stress and inflammatory response of AKI and hence advance the current therapies.

To conclude, the study suggests that the infusion of oxygen-filled PFPs helps to improve the level of renal oxygenation capacity, provides good renal protection and recovery during the reperfusion phase through preprotection, and presents a novel strategy for the clinical treatment of AKI.

In conclusion, our study developed a PFP emulsion that improves oxygen delivery in AKI, preserving renal function and reducing injury by modulating oxidative stress and inflammation. Though PFPs show promise as a therapeutic strategy for improving kidney function under hypoxic conditions, the study’s scope was limited to short-term effects, with long-term impacts and unknown toxicity. The renal protection mechanisms of PFPs are also undefined and merit deeper exploration.

## Ethical Approval

The authors are accountable for all aspects of the work in ensuring that questions related to the accuracy or integrity of any part of the work are appropriately investigated and resolved.

## Data Availability

The data that support the findings of this study are available from the corresponding authors upon reasonable request.
