# Supplementary material for: Perfluorocarbon Nanoparticles Loaded with Oxygen Alleviate Acute Kidney Injury via Ameliorating Renal Oxygenation Level
Source: Biomater Res. 2025 Apr 10;29:0181. doi: 10.34133/bmr.0181 (PMC11982615; doi:10.34133/bmr.0181)
Supplement: Supplementary 1 — Fig. S1 [file bmr.0181.f1.docx]

**Perfluorocarbon nanoparticles loaded with oxygen alleviate acute kidney injury via ameliorating renal oxygenation level**

Dasheng Li^1,2#^, Yisong Ju^3#^, Qingsong Ye^4#^, Yuanyuan Chang^3^, Chaoli An^1,2^, Beibei Liu^1,2^, Li Lu^1,2,3^*, Jinhui Wu^4^* and Xiaozhi Zhao^1,2,3^*

**Supplementary Figure 1**


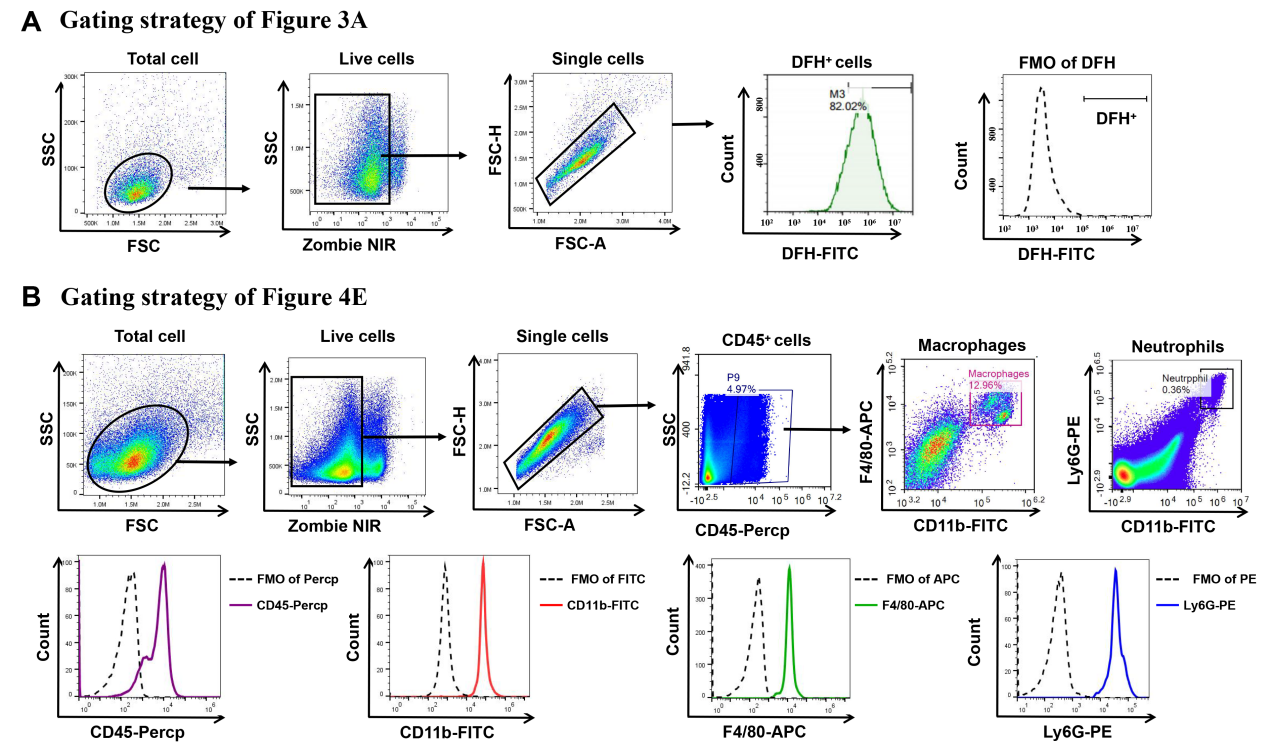


**Supplementary Figure 1:** (A) Gating strategy of Figure 3A, and Fluorescence Minus One (FMO) of DFH; (B) Gating strategy of macrophages and Neutrophils in mouse kidney, and FMO of CD45-Percp, CD11b-FITC, F4/80-APC and Ly6G-PE.
